# Phase 1 trial of dasatinib combined with afatinib for epidermal growth factor receptor- (EGFR-) mutated lung cancer with acquired tyrosine kinase inhibitor (TKI) resistance

**DOI:** 10.1038/s41416-019-0428-3

**Published:** 2019-03-18

**Authors:** Ben C. Creelan, Jhanelle E. Gray, Tawee Tanvetyanon, Alberto A. Chiappori, Takeshi Yoshida, Michael J. Schell, Scott J. Antonia, Eric B. Haura

**Affiliations:** 1 Thoracic Oncology, H. Lee Moffitt Cancer Center and Research Institute, 12902 Magnolia Dr., Tampa, FL 33612 USA; 2 Department of Medical Oncology, Kindai University Faculty of Medicine, 377-2, Ono-Higashi, Osakasayama, Osaka, 589-8511 Japan; 3 Department of Biostatistics and Bioinformatics, H. Lee Moffitt Cancer Center and Research Institute, 12902 Magnolia Dr., Tampa, FL 33612 USA

**Keywords:** Drug safety, Non-small-cell lung cancer, Non-small-cell lung cancer, Drug development, Cancer genomics

## Abstract

**Background:**

Bypass activation of Src family kinases can confer resistance to EGFR tyrosine kinase inhibitors (TKIs) based on preclinical models. We prospectively assessed the safety and clinical activity of dasatinib and afatinib in combination for patients with resistant EGFR-mutant lung cancer.

**Methods:**

An open-label, dose-escalation phase 1/2 trial (NCT01999985) with 2-stage expansion was conducted with 25 lung cancer patients. Dose expansion required activating EGFR mutations and progression following prior EGFR TKI.

**Results:**

Patients were 72% Caucasian and received median of 2 prior lines of therapy. Maximum-tolerated dose was 30 mg afatinib with 100 mg dasatinib. New or increased pleural effusions were observed in 56% of patients. No radiologic responses were observed, although several EGFR-mutant TKI-resistant patients (26%) had prolonged stable disease over 6 months. The combination reduced the EGFR mutation and T790M variant allele frequency in cell-free DNA (*p* < .05). Nonetheless, the threshold for futility was met, based on 6-month progression-free survival. For EGFR TKI-resistant patients, median progression-free survival was 3.7 months (95% confidence interval (CI), 2.3–5.0) and overall survival was 14.7 months (95% CI, 8.5–20.9).

**Conclusions:**

The combination had a manageable toxicity profile and in vivo T790M modulation, but no objective clinical responses were observed.

## Background

Despite progress in targeting epidermal growth factor receptor-mutant (EGFRm) non-small cell lung cancer (NSCLC), acquired resistance to EGFR tyrosine kinase inhibitors (TKIs) is inevitable. Common mechanisms include secondary EGFR mutations, such as T790M, bypass receptor tyrosine kinase activation, such as through MET amplification, or tumour reprogramming through epithelial–mesenchymal transition or transformation to small cell lung cancer. Another mechanism is through signalling from Src family kinases (SFKs). Constitutive activation SFKs may contribute to EGFR TKI resistance, in part through ligand-independent activation of c-met.^1^ By combining dasatinib with the irreversible EGFR TKI afatinib, we observed synergistic lethality in EGFRm TKI-resistant cell lines and significant tumour reduction in mice bearing EGFR-mutant xenografts harbouring the T790M mutation.^2^ In cell lines, dasatinib overcomes downstream kinase activation associated with T790M TKI resistance, including Src, Akt, and Erk.^3^ High levels of phospho-Src have been identified in tumours after EGFR TKI resistance has been reached.^4^ Loss of *PTEN* may also contribute to TKI resistance via downstream Src/Akt pathway activation.^5,6^ Overexpression of the Yes-associated protein *YAP* is associated with resistance to first-generation EGFR TKIs.^7^ Overexpression of the EGF-CFC (epidermal growth factor-Cripto-1/FRL-1/Cryptic) protein family member CRIPTO-1 has been identified to directly enable EGFR resistance through SRC activation^8^ and has been associated with primary TKI resistance among EGFRm patients.^9^ More recent studies using transposon mutagenesis assays identified the SFK member *YES1* as a mediator of resistance to all three generations of EGFR TKIs and conferred sensitivity to dasatinib.^10,11^ SFKs appear to sustain AKT and mitogen-activated protein kinase (MAPK) pathway signalling during osimertinib treatment, and combined dasatinib and osimertinib causes inhibition of cancer growth, apoptosis, and delay of acquired resistance in EGFRm animal studies.^12^ Therefore, disruption of the SFK pathway may still remain a viable method of overcoming TKI resistance, and further study of novel approaches is warranted.

Dasatinib is a potent inhibitor of several tyrosine kinase families, including the SFKs. In two phase 2 trials, the combination of dasatinib and erlotinib mediated tumour reductions in two EGFRm patients with acquired resistance, but overall response rates remained low.^4,13,14^ Based on our preclinical studies demonstrating improved in vivo efficacy of dasatinib and afatinib in T790M models of acquired resistance to first-generation EGFR TKI, we hypothesised that this combination would lead to durable disease control in EGFRm NSCLC patients with acquired TKI resistance. Therefore, we conducted a phase 1/2 trial with the primary objective of characterising the safety and clinical activity of dasatinib with afatinib in this population. We also hypothesised that assessment of cell-free DNA for EGFR mutations could serve as an additional readout of drug efficacy.

## Materials and methods

This was an open-label, single-centre, phase 1 study with a modified 3 + 3 dose-escalation design, followed by an expansion cohort with a 2-stage design (NCT01999985). The trial was approved by Liberty Institutional Review Board Inc., assurance number IRB00003411. For dose escalation, patients were required to have stage 4 NSCLC with progression after ≥1 standard therapy. For dose expansion, patients were required to have an activating EGFR exon 19 or 21 mutation, with disease progression after ≥1 TKI (gefitinib, afatinib, or erlotinib). On the basis of the concentrations observed to modulate Src in vivo, starting doses of 100 mg dasatinib and 30 mg afatinib once daily were chosen. T790M positive was defined as the presence of detectable allele in either tumour or plasma. Patient with pericardial or pleural effusion of grade 2 or higher were excluded. Patients were required to have RECIST evaluable disease. Enlargement of pre-existing pleural effusions by itself was interpreted cautiously, and not counted as a non-target progression without convincing evidence.

The sample size of the escalation cohort was powered to conclude the dose-limiting toxicity (DLT) rate was ≤33% if no DLTs were observed in 8 patients treated at the dose level, with 85% power and a 1-sided ɑ of 0.10. For the expansion cohort, the null hypothesis was that 26% of patients would be progression free at 6 months, and the alternative hypothesis was that true progression-free survival would be 6.6 months, e.g., a progression-free rate of 52%. Using one-sided binomial test with actual ɑ = 0.046, this design had 66% power with *n* = 14. For an expanded description of the methods involved in the study, please see Supplementary Material: Methods.

## Results

### Patient characteristics

**Fig. 1:**
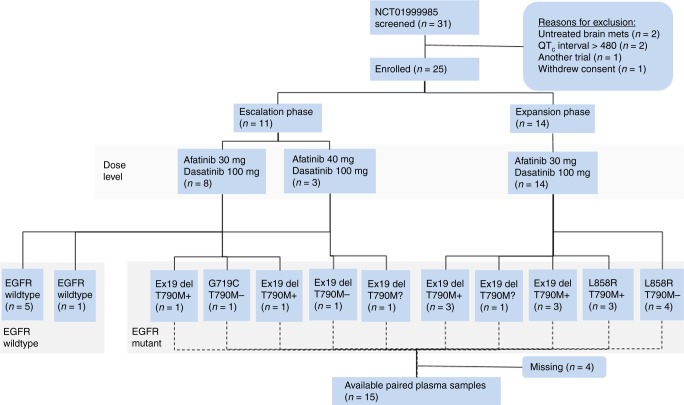
Diagram showing patient treatment assignment, baseline epidermal growth factor receptor (EGFR) status, and sample availability. All patients had first-line epidermal growth factor receptor tyrosine kinase inhibitor resistance. EGFR mutation status assessed by plasma or tissue test: T790M+ detected, T790M− not detected, T790M? unknown

Of the 31 patients screened, 25 were eligible and treated (Fig. 1). Patients had received a median of 2 (range, 1–5) prior systemic therapies for stage 4 NSCLC and had progressive measurable disease (Supplemental Table S1). Patients with an activating EGFR mutation had previously progressed on a first- or second-generation EGFR TKI. No patients had evidence of small cell transformation, and none had received prior third-generation TKIs, such as osimertinib.

### Safety and tolerability

Dose escalation proceeded through 2 dose levels of up to 40 mg afatinib with 100 mg dasatinib. Although no protocol-defined DLT was observed, we decided to deescalate after 3 patients were enrolled at the 40 mg dose level, due to the persistence of grade 2 diarrhoea despite optimal medical management in 2 patients. These two events were therefore recorded as DLTs for monitoring purposes.

**Fig. 2:**
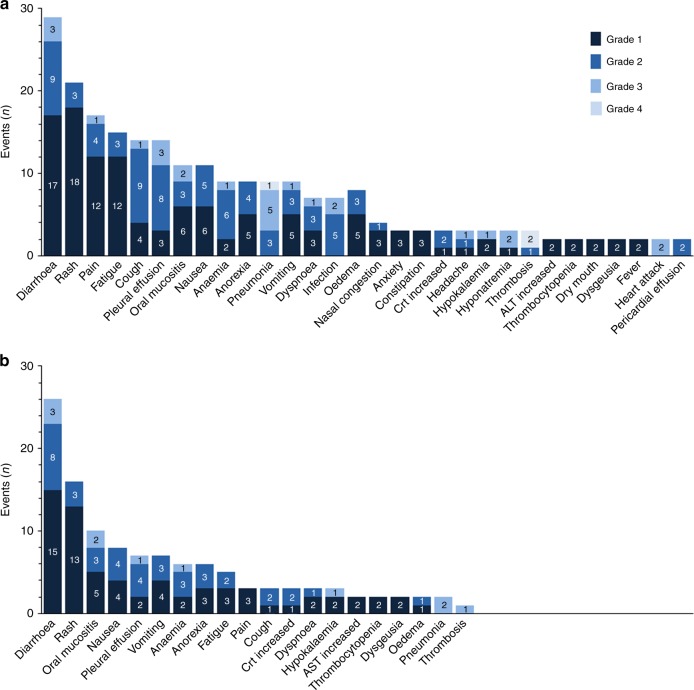
Graphs show all adverse events occurring more than once, and any event grade ≥2 in severity. Numbers of adverse events for each category are shown in the bars. **a** Total adverse events (*n* = 229 are shown). **b** Treatment-related adverse events (*n* = 117 are shown). ALT alanine transferase, Crt creatinine

Patients received a mean of 3.2 months of continuous oral drug exposure. Adverse events (AEs) for both dose levels in all patients are summarised together (Fig. 2). The most common drug-related AEs were diarrhoea (72%) and rash (64%) (Supplemental Table S2). The most common serious AEs, regardless of causality, were pneumonia (24%), diarrhoea (12%), and pleural effusion (12%). Although drug-related AEs were generally manageable, 24% of patients eventually required dose reductions or interruptions for the management of AEs.

New or increased pleural effusions occurred in 72% of patients on reimaging computed tomography (CT) scans (Supplemental Table S3). In many instances, it was difficult to ascertain whether effusions were attributable to dasatinib or lung cancer, since enlargement of pre-existing malignant effusions could be attributable to either cause. Symptomatic pleural effusions were managed with thoracentesis, corticosteroid tapers, or dose reductions of dasatinib to 50 mg daily, if recrudescence occurred.

No QTc prolongations on serial electrocardiograms were detected during the trial (Supplemental Fig. 1A). No decrease in mean left ventricular ejection fraction below normal levels (<50%) was detected (Supplemental Fig. 1B).

### Pharmacodynamics

**Fig. 3:**
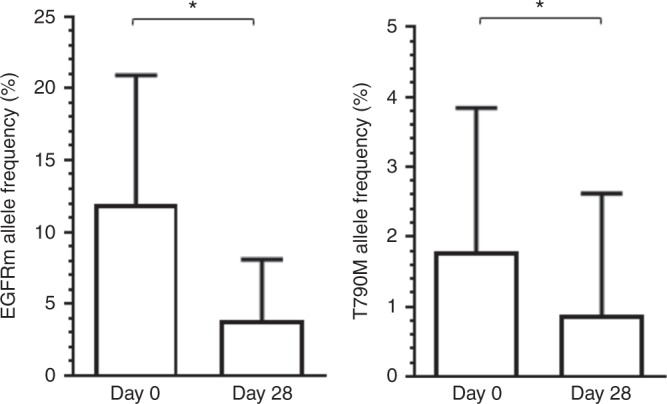
Decrease in mutant allele fraction from plasma cell-free DNA during afatinib–dasatinib treatment. Patients with activating epidermal growth factor receptor mutations had paired samples assessed by digital droplet polymerase chain reaction. Fifteen patients were evaluable. Bars represent mean values with 95% confidence intervals. *P < .05 by Wilcoxon matched-pairs signed-rank test

For patients with activating EGFRm with acquired TKI resistance, an overall decrease in EGFRm-variant allele frequency was observed during the course of treatment (Fig. 3a). The interpretation was limited by a high degree of deviation in variant allele frequencies between patients. Likewise, a decrease in T790M allele frequency was observed among patients with baseline detectable T790M (Fig. 3b). Samples for subsequent time points were not collected.

### Treatment outcomes and overall survival

**Fig. 4:**
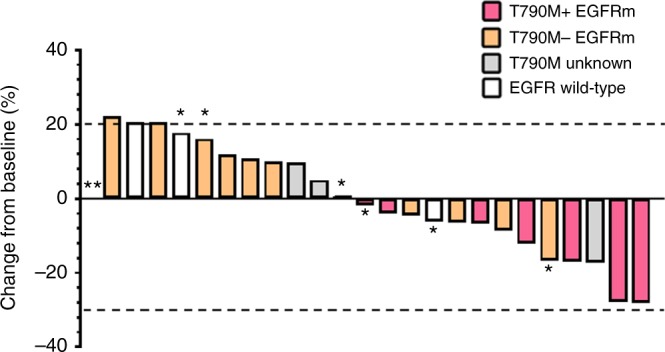
Waterfall plot shows best percentage changes in sums of target lesions compared to baseline. *Progression by non-target or new lesion. **Target non-evaluable at progression

**Fig. 5:**
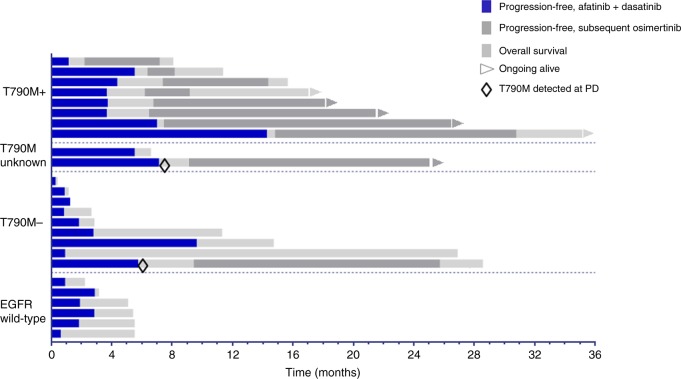
Swimmers plot shows individual progression-free and overall survival of afatinib–dasatinib patients by baseline epidermal growth factor receptor mutation status

No radiologic responses were observed, although several EGFRm TKI-resistant patients had stable disease as best response (Fig. 4). One patient with acquired T790M had stable disease lasting more than 12 months. The trial met the interim stopping threshold for futility based upon 6-month progression-free survival. For all EGFR TKI-resistant patients, median progression-free survival was 3.7 months (95% confidence interval (CI), 2.3–5.0). Median overall survival was 14.7 months (95% CI, 8.5–20.9) at a median follow-up of 25 months. EGFRm patients lacking detectable T790M at entry had worse overall survival than those with detectable T790M at entry, with a hazard ratio of 4.0 (95% CI, 1.3–13.1), *p* = .02. This may be partly attributable to subsequent osimertinib as the next line of therapy for patients who were T790M positive at study entry, as shown in Fig. 5. All deaths were attributable to progressive cancer. No ostensible clinical benefit was detected in the EGFR wild-type population, with a median progression-free survival of 1.8 months (95% CI, 0.7–3.0) and median overall survival of 5.1 months (95% CI, 2.4–7.8).

## Discussion

In this phase 1 trial, it was feasible to combine dasatinib with afatinib in advanced NSCLC at biologically active doses. Nonetheless, pleural effusion remained a prominent adverse effect, with most patients who had been effusion free at study entry exhibiting pleural effusions on their reimaging CT scans. The inclusion of patient with mild pleural effusions may have confounded the interpretation of this adverse effect. Nevertheless, our goal was to enrol representative patients, since malignant pleural effusions occur in almost 40% of metastatic NSCLC.^15^ Moreover, the clinical activity of the combination was limited and did not justify further investigation, especially since third-generation EGFR TKIs such as osimertinib have become available and demonstrate significant responses in a similar patient population.

This combination was observed to modestly decrease the fraction of plasma-mutant EGFR and T790M alleles at week 4. In contrast, complete clearance of plasma T790M occurred at week 6 among most patients on the *AURA* osimertinib trial and was associated with durable response.^16^ Although dasatinib has modest in vitro binding affinity to mutant EGFR kinases, T790M appears to cause steric hindrance with the chloro-methyl-phenyl ring of dasatinib.^17^ Nonetheless, dasatinib is hypothesised to suppress bypass tracks through SRC/AKT, rather than direct inhibition of the T790M EGFR kinase.

Despite promising preclinical activity, dasatinib is characterised by a short terminal half-life in plasma, which may constrain its inhibitory activity of SFKs in human tissue.^18^ To this end, the reported tolerability and efficacy of dasatinib as a single agent across multiple solid tumours has been unsatisfactory. Two phase 1/2 NSCLC trials of single-agent dasatinib at higher doses were closed due to a high frequency of AEs and a paucity of durable responses.^19,20^ Likewise, additional trials of dasatinib with erlotinib reported low or absent responses in patients with acquired EGFR TKI resistance.^13,14^ Finally, a phase 2 trial of dasatinib for inactivating BRAF mutations was closed due to a lack of efficacy amongst the study population.^21^ Therefore, a trial testing osimertinib with dasatinib in EGFRm NSCLC based upon baseline plasma or tumour CRIPTO-1 expression is ongoing.^22^ Additionally, YAP amplification has been proposed to confer resistance in up to 4% of EGFR TKI-resistant patients, which may implicate SFK as important in mediating resistance.^7^ Therefore, there may yet be a further role for SFK inhibitors in these narrowly defined molecular subgroups. In our study, tumour biopsies were not performed, and thus we could not detect phospho-Src, YES amplification, or Cripto-1 alterations within the tumour. Future studies of SFK in combination with EGFR targeting agents should focus on subsets of patients with likely mechanisms of resistance driven by SFK.

## Supplementary information

Table S1

Table S2

Table S3

Figure S1

Supplemental Methods Section

Figure 1S

## **Data availability:**

Raw data and materials generated during the study are available upon request to the author study team, thoraciccro@moffitt.org.
