# Supplementary material for: Phase 1 trial of dasatinib combined with afatinib for epidermal growth factor receptor- (EGFR-) mutated lung cancer with acquired tyrosine kinase inhibitor (TKI) resistance
Source: Br J Cancer. 2019 Mar 18;120(8):791–6. doi: 10.1038/s41416-019-0428-3 (PMC6474279; doi:10.1038/s41416-019-0428-3)
Supplement: Supplementary file 1 — Table S1 [file 41416_2019_428_MOESM1_ESM.doc]

| **Table S1.** Patient characteristics | | | |  |
| --- | --- | --- | --- | --- |
| Characteristic | Escalation (*n* = 11) | Expansion (*n* = 14) | Total (*n* = 25) |  |
| Age, years |  |  |  |  |
| Median (range) | 66 (53-77) | 68 (44-81) | 66 (44-81) |  |
| Gender, No. (%) |  |  |  |  |
| Female | 7 (63) | 8 (57) | 15 (60) |  |
| Lung cancer type, No. (%) |  |  |  |  |
| Adenocarcinoma | 9 (82) | 14 (100) | 23 (92) |  |
| Squamous  Uveal | 2 (18) | -- | 2 (8) |  |
| EGFR allele, No. (%) |  |  |  |  |
| Exon 19 deletion | 4 (36) | 7 (50) | 11 (44) |  |
| L858R substitution | -- | 7 (50) | 7 (28) |  |
| G719C substitution | 1 (9) | -- | 1 (4) |  |
| Wild-type | 6 (55) | -- | 6 (24) |  |
| AJCC *v*7 stage, No. (%) |  |  |  |  |
| IV | 11 (100) | 14 (100) | 25 (100) |  |
| Dose level, No. (%) |  |  |  |  |
| Afatinib 30 mg | 8 (73) | 14 (100) | 22 (88) |  |
| Afatinib 40 mg | 3 (27) | -- | 3 (12) |  |
| Prior lines of systemic therapy |  |  |  |  |
| Median, range | 3 (1-5) | 2 (1-3) | 2 (1-5) |  |
| Prior palliative radiation, No. (%) |  |  |  |  |
| Yes | 7 (63) | 10 (71) | 17 (68) |  |
| No | 4 (37) | 4 (29) | 8 (32) |  |
| Prior treated brain metastases, No. (%) |  |  |  |  |
| Yes | 3 (27) | 5 (36) | 8 (32) |  |
| Race, No. (%) |  |  |  |  |
| Caucasian | 11 (100) | 7 (50) | 18 (72) |  |
| Black | -- | 5 (36) | 5 (20) |  |
| Asian | -- | 2 (14) | 2 (8) |  |
| **EGFR mutant subgroup** | (*n* = 5) | (*n* = 14) | (*n* =19) |  |
| Baseline T790M, No. (%) |  |  |  |  |
| Detected | 2 (40) | 6 (42) | 8 (42) |  |
| Not detected | 2 (40) | 7 (50) | 9 (47) |  |
| Unknown | 1 (20) | 1 (7) | 2 (11) |  |
| Received prior TKI, No. (%) |  |  |  |  |
| Yes | 5 (100) | 14 (100) | 19 (100) |  |
| Prior EGFR TKI type, No. (%) |  |  |  |  |
| Erlotinib | 4 (80) | 13 (93) | 17 (89) |  |
| Gefitinib | -- | 1 (7) | 1 (5) |  |
| Afatinib | 1 (20) | -- | 1 (5) |  |
| Abbreviations: AJCC, American Joint Committee on Cancer v7;  All percentages are rounded and therefore may not exactly sum to one. Stage is assessed at enrollment. No patients received previous osimertinib or other 3^rd^ generation TKI. No patients had active brain metastases. | | | | |
