# Supplementary material for: Phase 1 trial of dasatinib combined with afatinib for epidermal growth factor receptor- (EGFR-) mutated lung cancer with acquired tyrosine kinase inhibitor (TKI) resistance
Source: Br J Cancer. 2019 Mar 18;120(8):791–6. doi: 10.1038/s41416-019-0428-3 (PMC6474279; doi:10.1038/s41416-019-0428-3)
Supplement: Supplementary file 2 — Table S2 [file 41416_2019_428_MOESM2_ESM.doc]

| **Table S2a.** Summary of Adverse Events (%), *n* = 25. | | | |  |  |
| --- | --- | --- | --- | --- | --- |
|  | Grade 1 | Grade 2 | Grade 3 | Grade 4 | All grades |
| Diarrhea | 40 | 20 | 12 | - | 72 |
| Rash | 52 | 12 | - | - | 64 |
| Pain | 36 | 20 | 8 | - | 64 |
| Fatigue | 36 | 12 | - | - | 48 |
| Cough | 16 | 36 | 4 | - | 56 |
| Pleural effusion | 12 | 32 | 12 | - | 56 |
| Oral mucositis | 12 | 8 | 8 | - | 28 |
| Nausea | 24 | 20 | - | - | 44 |
| Anemia | 8 | 24 | 4 | - | 36 |
| Anorexia | 20 | 16 | - | - | 36 |
| Pneumonia | - | 12 | 20 | 4 | 36 |
| Vomiting | 20 | 12 | 4 | - | 36 |
| Dyspnea | 12 | 12 | 4 | - | 28 |
| Infection | - | 20 | 8 | - | 28 |
| Edema | 20 | 12 | - | - | 32 |
| Nasal congestion | 12 | 4 | - | - | 16 |
| Anxiety | 12 | - | - | - | 12 |
| Constipation | 12 | - | - | - | 12 |
| Crt increased | 4 | 8 | - | - | 12 |
| Headache | 4 | 4 | 4 | - | 12 |
| Hypokalemia | 8 | - | 4 | - | 12 |
| Hyponatremia | 4 | - | 8 | - | 12 |
| Thrombosis | - | 4 | - | 8 | 12 |
| ALT increased | 8 | - | - | - | 8 |
| Thrombocytopenia | 8 | - | - | - | 8 |
| Dry mouth | 8 | - | - | - | 8 |
| Dysgeusia | 8 | - | - | - | 8 |
| Fever | 8 | - | - | - | 8 |
| Heart attack | - | - | 8 | - | 8 |
| Pericardial effusion | - | 8 | - | - | 8 |
| Abbreviations: ALT; alanine transferase; Crt, creatinine. ;  All percentages are rounded and therefore may not exactly sum to one. Shown is worst-grade adverse event in two or more patients, and all grade 3, 4, 5 adverse events, of any causality, except for death from cancer. | | | | |  |

| **Table S2b.** Summary of Drug-related Adverse Events (%), *n* = 25. | | | |  |  |
| --- | --- | --- | --- | --- | --- |
|  | Grade 1 | Grade 2 | Grade 3 | Grade 4 | All grades |
| Diarrhea | 36 | 12 | 12 | - | 60 |
| Rash | 40 | 12 | - | - | 52 |
| Oral mucositis | 12 | 8 | 8 | - | 40 |
| Nausea | 16 | 16 | - | - | 32 |
| Pleural effusion | 8 | 16 | 4 | - | 28 |
| Vomiting | 16 | 12 | - | - | 28 |
| Anemia | 8 | 12 | 4 | - | 24 |
| Anorexia | 12 | 12 | - | - | 24 |
| Fatigue | 12 | 8 | - | - | 20 |
| Pain | 12 | - | - | - | 12 |
| Cough | 4 | 8 | - | - | 12 |
| Crt increased | 4 | 8 | - | - | 12 |
| Dyspnea | 8 | 4 | - | - | 12 |
| Hypokalemia | 8 | - | 4 | - | 12 |
| AST increased | 8 | - | - | - | 8 |
| Thrombocytopenia | 8 | - | - | - | 8 |
| Dysgeusia | 8 | - | - | - | 8 |
| Edema | 4 | 4 | - | - | 8 |
| Pneumonia | - | - | 8 | - | 8 |
| Thrombosis | - | - | 4 | - | 4 |
| Abbreviations: ALT; alanine transferase; Crt, creatinine.  All percentages are rounded and therefore may not exactly sum to one. Shown is worst-grade adverse event of two counts or more, and all grade 3, 4, 5 adverse events, of “possible”, “probable”, or “definite” attribution to study drug. | | | | |  |
