# Supplementary material for: Phase 1 trial of dasatinib combined with afatinib for epidermal growth factor receptor- (EGFR-) mutated lung cancer with acquired tyrosine kinase inhibitor (TKI) resistance
Source: Br J Cancer. 2019 Mar 18;120(8):791–6. doi: 10.1038/s41416-019-0428-3 (PMC6474279; doi:10.1038/s41416-019-0428-3)
Supplement: Supplementary file 3 — Table S3 [file 41416_2019_428_MOESM3_ESM.doc]

| **Table S3.** Pleural Effusion / Diarrhea characteristics (n, %) | | | |  |
| --- | --- | --- | --- | --- |
| Characteristic | 30 mg afatinib 100 mg dasatinib  (*n* = 22) | 40 mg afatinib 100 mg dasatinib  (*n* = 3) | Total (*n* = 25) |  |
| **Diarrhea** |  |  |  |  |
| Any grade | 11 (50) | 3 (100) | 14 (56) |  |
| Required loperamide /diphenoxylate | 10 (45) | 3 (100) | 13 (52) |  |
| Required parenteral hydration | - | 1 (33) | 1 (4) |  |
| **Pleural Effusion** |  |  |  |  |
| Pre-existing effusion | 13 (59) | 1 (33) | 14 (56) |  |
| Increased on-treatment | 12 (55) | - | 12 (48) |  |
| New effusion on-treatment | 5 (23) | 2 (66) | 6 (24) |  |
| Required thoracentesis on-treatment | 3 (14) | 1 (33) | 4 (16) |  |
| Malignant effusion by cytology | 9 (41) | - | 9 (36) |  |
| All percentages are rounded and therefore may not exactly sum to one.  Shown is the total numbers of patients with each condition, by dose level. | | | | |
