# Supplementary figures and images for: Phase 1 trial of dasatinib combined with afatinib for epidermal growth factor receptor- (EGFR-) mutated lung cancer with acquired tyrosine kinase inhibitor (TKI) resistance
Source: Br J Cancer. 2019 Mar 18;120(8):791–6. doi: 10.1038/s41416-019-0428-3 (PMC6474279; doi:10.1038/s41416-019-0428-3)

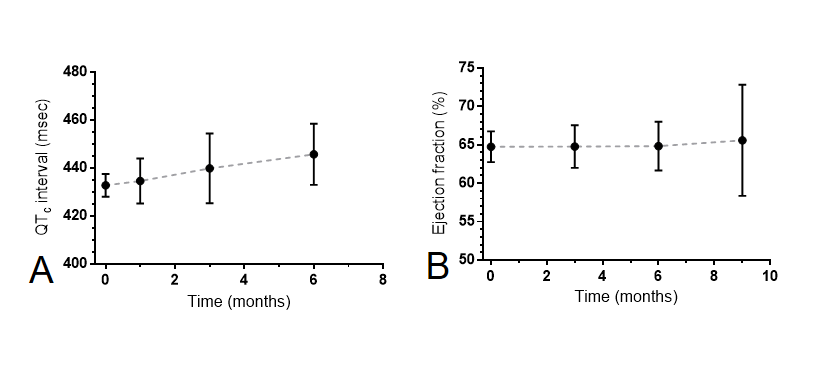

Supplement: Supplementary file 4 — Figure S1 [file 41416_2019_428_MOESM4_ESM.tif]
