# Supplementary material for: Phase 1 trial of dasatinib combined with afatinib for epidermal growth factor receptor- (EGFR-) mutated lung cancer with acquired tyrosine kinase inhibitor (TKI) resistance
Source: Br J Cancer. 2019 Mar 18;120(8):791–6. doi: 10.1038/s41416-019-0428-3 (PMC6474279; doi:10.1038/s41416-019-0428-3)
Supplement: Supplementary file 5 — Supplemental Methods Section [file 41416_2019_428_MOESM5_ESM.docx]

**Phase I trial of dasatinib combined with afatinib for epidermal growth factor receptor- (EGFR-) mutated lung cancer with acquired tyrosine kinase inhibitor (TKI) resistance**

**SUPPLEMENTAL METHODS**

**Trial design**

This trial was conducted from 2014 to 2016 at the H. Lee Moffitt Cancer Center (Tampa, FL), after approval by Liberty Institutional Review Board, Federal-Wide Registration #008679, Study #12.09.0006. It was performed in accordance with the Declaration of Helsinki and the International Conference on Harmonization Good Clinical Practice guidelines. Informed consent was obtained from all patients. The primary objectives were to characterize the safety and tolerability of afatinib and dasatinib combination treatment, determine the recommended phase II dose, analyse pharmacodynamics, and examine preliminary clinical activity in patients with epidermal growth factor receptor- (EGFR-) tyrosine kinase inhibitor (TKI) resistance.

**Treatment**

Starting on day 1, 30 mg Afatinib were administered orally daily, with a cycle every 28 days. In the dose-escalation phase, 100 mg dasatinib was started on day 8 to allow for toxicity evaluation, and in the dose-expansion phase, it began concurrently with afatinib on day 1. The dosing would increase to next level of afatinib 40 mg daily with dasatinib 100 mg daily, and proceed to final level of afatinib 40 mg daily with dasatinib 100 mg twice daily if no dose-limiting toxicities were seen, in a modified Fibonacci 3 + 3 design.

**Eligibility**

Patients aged 18 years or older, with histologically confirmed stage 4 non-small cell lung cancer who had experienced disease progression after ≥ 1 standard line of therapy were eligible for the dose escalation cohort of this trial. Patients with EGFR mutation were required to have progressed on at least 1 prior EGFR TKI. Other inclusion criteria included: toxicity from prior therapy resolved to grade ≤ 1, an Eastern Cooperative Oncology Group (ECOG) performance status ≤ 1, and adequate organ function. Patients with stable treated brain metastases were eligible.

Significant exclusion criteria included untreated brain metastases, requirement for supplemental oxygen, left ventricular ejection fraction < 50%, QTc interval on electrocardiogram at screening of > 470 msec for men or > 480 msec for women. Patients taking inhibitors of p-glycoprotein were also excluded.

**Definitions of DLT and MTD**

Dose-limiting toxicity (DLT) was determined based on toxicities observed in the first cycle that the investigator assessed as being possibly related to either drug. Patients were required to complete a mandatory DLT evaluation period of ≥ 28 days and all enrolled patients were included in the safety analysis. DLTs were defined as any hepatobiliary disorders grade ≥ 2 or any grade ≥ 3 nonhepatobiliary toxicity of any duration. Grade 3 nausea or vomiting were only considered DLTs if they occurred despite optimal medical management. Total bilirubin, aspartate aminotransferase, and alanine aminotransferase were considered DLTs if > 8X upper limit of normal (ULN), > 5 X ULN for over 2 weeks, or > 3X ULN with symptoms. The MTD was defined as the highest dose at which ≤ 1 of 6 DLTs (< 33%) patients experienced DLT at that dose level.

**Safety and Efficacy**

Clinical and laboratory assessments were conducted at baseline and weekly thereafter. Safety assessments included medical history, physical examination, electrocardiogram, hematology, urinalysis, and biochemistry. Adverse events were graded using Common Toxicity Criteria for Adverse Events version 4.0. Tumour assessments were performed every 2 cycles using Response Evaluation Criteria in Solid Tumour guidelines version 1.1.(1) Patients continued to be followed for toxicity for 30 days after drug discontinuation.

**Rationale for Omission of Pharmacokinetic Profiling**

The available literature did not provide theoretical reason to expect that afatinib significantly affects the metabolism or elimination of dasatinib. Dasatinib is metabolized by CYP3A4 (primarily), flavin-containing mono-oxygenase-3 (FOM-3), and uridine diphosphate-glucuronosyltransferase (UGT). Afatinib is not known to inhibit either of these pathways. Afatinib is primarily excreted unchanged as parent compound in the faeces, although a minor fraction is metabolized by P-gp mechanisms. It is not a significant substrate of P450 enzymes such as CYP 3A4, nor is it metabolized by FOM-3. (2) Therefore, it was deemed unlikely that dasatinib could affect the pharmacokinetic profile of afatinib. Dasatinib has been demonstrated to be a substrate of human P-glycoprotein (P-gp) in Madin-Darby canine kidney (MDCK) II cells. (3) However, dasatinib exhibits high intrinsic permeability, and thus a meaningful impact of P-gp on its intestinal absorption upon oral administration was unlikely. (BMS-354825 Investigator Brochure v13.0 11-3-10, pg 25). Dasatinib is not an inhibitor of P-gp in Caco-2 cells and was not expected to alter the absorption and distribution characteristics of compounds that are P-gp substrates.

**Pharmacodynamics**

Plasma levels of mutant EGFR alleles were determined from blood samples collected on days 1 and 28 prior to dosing. Plasma was separated and stored at −70°C until analysis. Pharmacodynamic samples were sent to Biodesix for analysis and processed in a single batch. Cell-free DNA was extracted from plasma QIAmp circulating nucleic acid Kit. DNA was quantified with Qubit DNA HS Assay Kit. Droplets were prepared, generated, and analyzed with cut-off of > 0. 2D. Quantaplots were visually inspected for artifacts, and analytic positive control and no-template controls were run with each batch. Replicate reactions were averaged, and wild-type and mutant alleles were quantified. Labels were assigned to each result (detected, not detected, indeterminate).

**Rationale for Selection of Starting Dose**

Afatinib 40 mg daily is the approved starting dose for the treatment of patients with advanced EGFR mutant NSCLC. However, in the phase III LUX-Lung 3 trial, dose reductions occurred in 53% (122/229) of patients; the majority (86%) within the first 6 months of treatment. (4) Median progression-free survival was 11.3 months in patients who dose-reduced during the first 6 months of treatment, *vs* 11.0 months in patients who did not (HR = 1.25 [95% CI, 0.91–1.72]). Similar findings favouring progression-free survival in the 30 mg dose reduction group was observed in the phase III LUX-Lung 7 trial. (5) Therefore, a starting dose of 30 mg afatinib appeared to be justified.

A previous trial conducted at the primary institution demonstrated that dasatinib 100 mg daily did achieve reduction in phospho-Src-Tyr^416^ immunohistochemistry levels in the bone marrow compartment of patients with relapsed myelodysplastic syndrome. (6) Previous trials had confirmed the dasatinib 100 mg daily dose was clinically efficacious in chronic myeloid leukemia. (7) Therefore, this starting dasatinib dose was selected.

**Statistical Analyses**

Categorical variables were compared using Fisher’s exact test, and Wilcoxon rank sum was used for continuous variables. Where appropriate, changes between paired timepoints, such as QTc and BNP measurements, were assessed using Wilcoxon signed rank test. Median survival was estimated by Kaplan-Meier method and confidence intervals were reported. Follow-up was calculated by reverse Kaplan-Meier method.

Statistical analysis was performed using Prism 6.0 (La Jolla, CA, USA) and SPSS 17.0 (SPSS Inc., Chicago IL) software. Gene data were analyzed with Quanta (Systat, Inc., San Jose, CA).

1. Eisenhauer E, Therasse P, Bogaerts J, Schwartz L, Sargent D, Ford R, et al. New response evaluation criteria in solid tumours: revised RECIST guideline (version 1.1). European Journal of Cancer. 2009;45(2):228-47.

2. Wind S, Schmid M, Erhardt J, Goeldner R-G, Stopfer P. Pharmacokinetics of afatinib, a selective irreversible ErbB family blocker, in patients with advanced solid tumours. Clinical pharmacokinetics. 2013;52(12):1101-9.

3. Christopher LJ, Cui D, Wu C, Luo R, Manning JA, Bonacorsi SJ, et al. Metabolism and disposition of dasatinib after oral administration to humans. Drug Metabolism and Disposition. 2008.

4. Yang JC-H, Ahn M-J, Dickgreber NJ, Halmos B, Hirsh V, Hochmair MJ, et al. Influence of dose adjustment on afatinib safety and efficacy in patients (pts) with advanced EGFR mutation-positive (EGFRm+) non-small cell lung cancer (NSCLC). American Society of Clinical Oncology; 2015.

5. Hirsh V, Yang JC-H, Tan E-H, O'Byrne K, Zhang L, Boyer MJ, et al. First-line afatinib (A) vs gefitinib (G) for patients (pts) with EGFR mutation positive (EGFRm+) NSCLC (LUX-Lung 7): Patient-reported outcomes (PROs) and impact of dose modifications on efficacy and adverse events (AEs). American Society of Clinical Oncology; 2016.

6. Duong VH, Jaglal MV, Zhang L, Kale V, Lancet JE, Komrokji RS, et al. Phase II pilot study of oral dasatinib in patients with higher-risk myelodysplastic syndrome (MDS) who failed conventional therapy. Leukemia research. 2013;37(3):300-4.

7. Shah NP, Kim D-W, Kantarjian H, Rousselot P, Llacer PED, Enrico A, et al. Potent, transient inhibition of BCR-ABL with dasatinib 100 mg daily achieves rapid and durable cytogenetic responses and high transformation-free survival rates in chronic phase chronic myeloid leukemia patients with resistance, suboptimal response or intolerance to imatinib. haematologica. 2010;95(2):232-40.
