# Supplementary material for: Phase 1 trial of dasatinib combined with afatinib for epidermal growth factor receptor- (EGFR-) mutated lung cancer with acquired tyrosine kinase inhibitor (TKI) resistance
Source: Br J Cancer. 2019 Mar 18;120(8):791–6. doi: 10.1038/s41416-019-0428-3 (PMC6474279; doi:10.1038/s41416-019-0428-3)
Supplement: Supplementary file 6 — Figure 1S [file 41416_2019_428_MOESM6_ESM.docx]

Figure 1S. Cardiovascular safety parameters during afatinib-dasatinib treatment. (A) Corrected QT interval by electrocardiogram over time. (B) Left ventricular ejection fraction assessed by multigated acquisition scan over time. Circles represent mean values with 95% confidence intervals.
